# A hidden curriculum for environmental topics in medical education: Impact on environmental knowledge and awareness of the students

**DOI:** 10.3205/zma001609

**Published:** 2023-05-15

**Authors:** Patrick Straßer, Michael Kühl, Susanne J. Kühl

**Affiliations:** 1 University of Ulm, Institute of Biochemistry and Molecular Biology, Ulm, Germany

**Keywords:** environmental communication, climate communication, medical education, environmental knowledge, environmental awareness

## Abstract

**Objective:**

Climate change constitutes a major challenge. The higher education sector plays an important role in regard to climate change and the adaptation to its consequences. Various approaches toward the integration of environmental subject areas to higher education teaching have already been described in other studies, but there is a lack of data supporting the effectiveness of these approaches in changing not only the environmental knowledge of students, but also their awareness. To address this, the present study tracked whether student attitudes about the environment could be changed by implicitly addressing medically relevant environmental topics as part of an online seminar.

**Methods:**

Second semester students of molecular medicine attending a mandatory 14-hour online seminar, which was required to obtain additive key qualifications and which consisted of independent study phases as well as online class meetings, were divided into two groups: the intervention group (IG, n=27, thereof 20 in the pretest and 21 in the posttest) was exposed to medically relevant environmental topics, while the comparison group (CG, n=26, thereof 22 in the pretest and 21 in the posttest) was exposed to general, non-environmental medical topics. Surveys were conducted with standardized questionnaires before and after the seminar in order to study the influence on the students’ environmental knowledge, awareness and other personal attitudes.

**Results:**

While the seminar did not significantly change the environmental awareness in either group, the environmental knowledge of the IG was significantly increased by the group’s exposure to environmental topics. In addition, the IG assessed its own environmental awareness regarding sustainable working methods in a laboratory as significantly higher after the seminar than the CG did, and some students of the IG had become more interested in issues relating to sustainability.

**Conclusion:**

The approach used to communicate environmental content mainly increased the environmental knowledge of students and piqued the interest of some students in climate-related and environmental topics. However, it was not possible to change deeper personal attitudes about environmental awareness, especially everyday behavior.

## Zusammenfassung

**Zielsetzung:**

Der Klimawandel stellt eine große Herausforderung dar. Bei Klimaschutz und Anpassung an die Folgen des Klimawandels kommt dem Hochschulsektor eine bedeutende Rolle zu. Verschiedene Ansätze zur Integration von Umweltthemen in die universitäre Lehre sind bereits beschrieben, allerdings mangelt es an Daten zur Effektivität der Methoden hinsichtlich Veränderungen im Umweltwissen und Umweltbewusstsein bei den Studierenden. In der vorliegenden Studie wurde deswegen untersucht, ob sich die umweltbezogenen Haltungen von Studierenden durch die implizite Beschäftigung mit medizinisch relevanten Umweltthemen innerhalb eines Online-Seminars verändern lassen.

**Methodik:**

Studierende der Molekularen Medizin im zweiten Fachsemester wurden in einem 14-stündigen Online-Pflichtseminar zum Erwerb additiver Schlüsselqualifikationen, welches selbstorganisierte Phasen sowie Online-Präsenz-Termine umfasste, in zwei Gruppen eingeteilt: die Interventionsgruppe (IG, n=27, davon 20 im Prätest und 21 im Posttest) sollte sich mit medizinisch relevanten Umweltthemen beschäftigen, die Vergleichsgruppe (VG, n=26, davon 22 im Prätest und 21 im Posttest) mit allgemeinmedizinischen Themen. Um Einflüsse auf Umweltwissen, Umweltbewusstsein sowie weitere persönliche Aspekte zu untersuchen, wurden vor und nach dem Seminar Befragungen mittels standardisierter Fragebögen durchgeführt.

**Ergebnisse:**

Durch das Seminar wurde das Umweltbewusstsein in beiden Gruppen nicht signifikant verändert, wohingegen das Umweltwissen der IG durch die Beschäftigung mit Umweltthemen signifikant gesteigert werden konnte. Darüber hinaus schätzte die IG ihr Umweltbewusstsein zu nachhaltigem Arbeiten im Labor nach dem Seminar deutlich besser als die VG ein und das Interesse für Nachhaltigkeitsthemen wurde bei einigen Studierenden der IG geweckt.

**Schlussfolgerung:**

Mit dem angewendeten Ansatz zur Umweltkommunikation konnte hauptsächlich das Umweltwissen der Studierenden erhöht und das Interesse einiger für Klima- und Umweltthemen geweckt werden. Tiefergreifende persönliche Haltungen bezogen auf das Umweltbewusstsein und insbesondere das Alltagsverhalten konnten nicht beeinflusst werden.

## 1. Introduction

Curbing the global rise in temperature and coping with the consequences of climate change are going to be some of the greatest challenges in the coming years and decades. Climate change has global and regional impacts such as droughts, extreme weather events or a changed ecosystem composition [1]. In addition, consequences for the human body and psyche are becoming apparent [2], causing the World Health Organization to refer to climate change as the greatest health threat of the 21^st^ century [3]. All in all, climate change holds enormous potential for social conflict [1].

The Paris Climate Agreement was adopted in 2015 in order to slow global warming to well below 2°C (3.6°F) of the pre-industrial era rate [4]. Article 12 of this agreement calls for, among other things, education improvements and access to information about climate change. This includes university courses to provide future leaders and decision-makers with an awareness of the problem and the knowledge needed to address it. In addition, the transfer of scientific knowledge to society is to be regarded as a responsibility of the science. Accordingly, the higher education sector and university education have a crucial role to play in climate protection and the adaptation to the consequences of climate change.

The healthcare sector is a significant climate change contributor, responsible for annual greenhouse gas emissions averaging 4.4% of the total global emissions [5], and in some countries up to 10% [6]. Furthermore, several million tons of harmful and climate-damaging plastics are produced annually in (medical) research laboratories [7]. Plastics are so rich in emissions during their life cycle that they account for about 4.5% of total global greenhouse gas emissions [8]. Once they enter the environmental cycle, they do not decompose, which results in a negative impact on individuals, populations and biodiversity [9]. Current efforts to minimize emissions from the healthcare sector include the restructuring of supply chains and mobility in a climate-friendly manner, the establishment of a circular economy instead of disposable products and a switch to a sustainable energy management in healthcare facilities [10]. In order for future practicing and researching physicians to be able to meet the goal of a sustainable healthcare sector, they must be increasingly educated and sensitized on topics relating to climate change and environmental pollution, among others, through a target group-specific teaching content.

In order to meet the need for integrating environmental and climate topics into the teaching, especially their inclusion in courses and curricula [11], various options are available for either embedding these topics into existing courses or creating entirely new courses. A wide variety of approaches are being implemented, from integrating environmental topics into a single event (“piggybacking”) or into an entire course (“mainstreaming”), to designing new modules (“specializing”) and new transdisciplinary courses (“connecting"). “Piggybacking” can be a time- and resource-efficient starting point for further development towards broad syllabi and interdisciplinary environmental education [12].

However, a recent study conducted in Heidelberg, Germany shows that students in their final year of study are aware of the effects of climate change, but are not sufficiently aware of their social communication and prevention responsibilities [13]. This gap between knowledge and action is a well-documented problem [14], [15], [16] and how to close this gap is a crucial question for the future.

**Objective: **The purpose of the present study was to study whether it is possible to change the environmental knowledge, awareness and behavior of students of molecular medicine of the Medical Faculty Ulm by implicitly educating them about medically relevant environmental topics as part of the seminar “Presentation and Moderation Techniques” that is required to obtain additive key qualifications. To this end, the content of the existing seminar was adapted without changing the original learning objectives for the course published for the students. The hidden curriculum approach was deliberately chosen as a method to bring about possible changes in the students’ attitude without having to restructure an existing course, which is both time-consuming and expensive.

## 2. Methods and implementation

### 2.1. Seminar description

**Figure 1:**
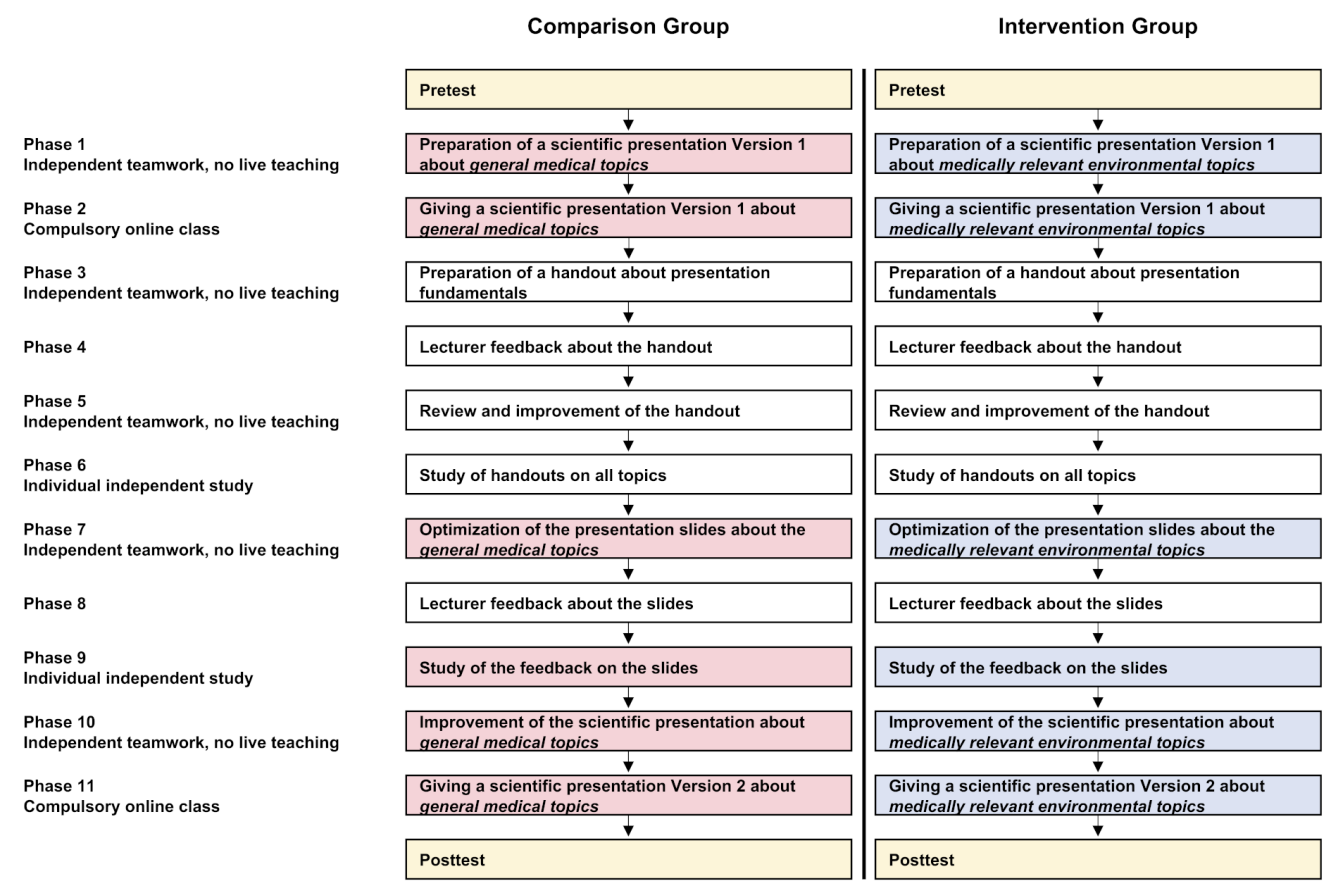
Study design and phases of the seminar titled “Presentation and Moderation Techniques” The phases relevant to the study are color-coded. Yellow: student survey. Red: general medical content. Blue: medically relevant environmental content (intervention). Categories asked about in the pretest and the posttest: Environmental knowledge, environmental affect, environmental cognition, environmental behavior, and seminar- and student-specific aspects; additional sociodemographic information in the pretest. During the entire seminar, the comparison and intervention groups were strictly separated, so that the presentations on the general medical and medically relevant environmental topics were given exclusively to the comparison and intervention groups, respectively.

The present study was conducted in connection with the “Presentation and Moderation Techniques” seminar, which was offered during the summer semester (SS) 2021 at the University of Ulm. It is a course for teaching interdisciplinary and practical competencies (additive key qualifications), which forms an explicit component of this Bachelor's program. Due to the Coronavirus pandemic, this seminar that is mandatory for students of molecular medicine in the second semester and takes a total of 14 hours extended over the entire semester (one semester hour per week) was offered as an online course. In 11 phases, consisting of independent study tasks (individually or in teams) as well as online presentations that were presented during scheduled online meetings, the participants learned in theory and practice of how to prepare and present scientific presentations. On the basis of presentation slides that were provided about specific topics, small groups (5-6 students per group) were asked to work on a presentation, which was then presented by one person of the group during a scheduled online meeting as part of a first round of presentation. After receiving feedback from students and lecturers and working on the basics of a scientific presentation, the students then incorporate the feedback to improve on their slides. For this purpose, the students were allowed to modify the graphics, structure and content of the presentations, but were required to stick to the topic of the first version. Finally, the presentations were given within the context of a short lecture (second round of presentations) (see figure 1 (Fig. 1)).

The learning objectives for the seminar were that students will be able to:

1. structure the content of a (scientific) presentation in a manner that makes it easy to understand,
2. use media and visuals in a targeted manner,
3. master general presentation fundamentals, and
4. discuss (scientific) presentations, papers and studies.

#### 2.2. Group classification and content of the study

When registering for the course, all 53 seminar participants were able to sign up for one of two groups without being aware of the course and study structure, the learning objectives or the content covered in their respective groups. The lecturer had no influence on this selection. The presentation topics, which the students worked on in independent study groups consisting of 5-6 persons, were assigned based on students’ names in alphabetical order.

In order to study whether environmental knowledge and awareness can be influenced by slide sets addressing medically relevant environmental topics, the intervention group (IG) with 27 participants and 5 teams of 5-6 persons each was asked to work on presentations about the following topics:

1. Plastic – harmful to health, yes or no?
2. The impact of climate change on mental and physical health
3. The impact of health care on global warming
4. Plastic consumption in research laboratories – is all of the consumption necessary?
5. What does the Corona pandemic have to do with species extinction?

In contrast, the following general medical topics were assigned to the comparison group (CG) with 26 students and 5 teams of 5-6 people each:

1. Should everyone automatically be an organ donor?
2. Compulsory vaccination – pros and cons
3. Are all the Corona vaccines good?
4. Online consultations: a good alternative to the traditional doctor's visit?
5. The influence of the pharmaceutical industry on the health care system

Not all seminar participants took part in the two surveys. Hence, the pretest was carried out with n=20 students (IG) and n=22 (CG) and the posttest with n=21 students (both groups).

#### 2.3. Data collection and analysis

Data was collected before the start of the seminar and announcement of the presentation topics (pretest), as well as after the end of the seminar (posttest) within a two-day time window each by means of online questionnaires (Tivian XI GmbH, EFS Survey, version 21.2). Questionnaires were completed on a voluntary and anonymous basis. For the study, the participants were each invited in an email sent to their university e-mail address. For this purpose, all students received an invitation link from the lecturer in accordance with the group they had been assigned to. As a participation incentive, “a little surprise” (a snack) was promised. To get this surprise, two solution words were listed on the last page of both surveys. This procedure was communicated at the beginning of the seminar and the students were asked to submit the solution words to be able to pick up the surprise.

Due to the sample size of n<30 in either group, the statistical procedure used was the nonparametric Wilcoxon-Mann-Whitney rank sum test. Differences with a p-value <0.05 were considered significant. All analyses were performed using the IBM SPSS Statistics for Macintosh software, version 28.0.1.0 (IBM Corp.).

#### 2.4. Questionnaire

In order to determine potential changes in students as a result of the environmental intervention (slide sets about medically relevant environmental topics), a questionnaire was developed specifically for this study (see attachment 1 ).

For a group comparison, socio-demographic information about the participants such as age, gender, previous education and environmental commitment was collected as part of the pretest.

In order to assess student attitudes about the environment, students were asked questions about the following environmental awareness subcategories: environmental affect (emotionally charged statements), environmental cognition (factual statements) and environmental behavior (everyday behavior). Participants were asked to rate 8 statements on a Likert-type scale ranging from 1 (=strongly disagree) to 6 (=strongly agree). The multidimensional environmental awareness classification and some of the questions were taken from surveys conducted by the German Federal Ministry for the Environment, Nature Conservation and Nuclear Safety (BMU) [17], the German Federal Environment Agency (UBA) [18] and the German Federal Agency for Civic Education (bpb) [19].

In addition, there were further questions relating to the seminar and the student. These were intended to provide information about personal and professional topics.

To ascertain student knowledge about the environment, 20 knowledge-related questions were asked in a multiple-choice format according to type A_pos_ (one correct answer out of five possible answers). Ten of these questions related to general medical lecture topics, and the remaining ten covered medical topics relating to the environment. There were two questions for each of the presentation topics, so that each individual presentation topic as well as the content of both groups were equally represented.

For validation purposes, the standardized questionnaire was put through four feedback loops. Ten employees and doctoral students as well as three experts in the field of teaching research participated. A pilot was conducted in a previous study [20]. Based on the results of that study, the answer formats were simplified, the environmental awareness subcategories were designed in a more balanced manner and the number of knowledge questions was increased.

#### 2.5. Ethics

The project was submitted to the ethics committee of the University of Ulm. The described educational research project was deemed not to require support or assistance. The anonymity of all data was guaranteed at all times.

## 3. Results

### 3.1. Intervention and comparison group socio-demographically comparable

In the pretest, no significant differences were found with regard to the average age (CG: 20.27; IG: 20.50) as well as the gender distribution (CG: 81.8% female; IG: 85.0% female) and environmental commitment (CG: 4.50%; IG: 5.00%) (see table 1 (Tab. 1)). In addition, when looking at the remaining 33 statements queried in the pretest about environmental affect, environmental cognition, environmental behavior, and seminar- and student-specific aspects, only one significant difference in the statement about meat consumption stands out when comparing the two groups (CG: 1.68, IG: 2.50, p<0.05, see table 2 (Tab. 2)). Thus, the CG and IG are characterized by matching basic requirements and can be considered comparable from a socio-demographic perspective.

#### 3.2. Effects on environmental awareness

There were no significant changes between pretest and posttest in either the CG or the IG with regard to environmental behavior, environmental affect or environmental cognition (see table 2 (Tab. 2), table 3 (Tab. 3) and table 4 (Tab. 4)). Both groups provided environmentally aware responses for the last two categories in the pretest. These high agreement values for the individual statements are again evident in the posttest. The following statements with average agreement values of at least 5.00 (out of 6) are mentioned by way of example: “I am worried about the environmental conditions in which future generations will probably have to live.” (environmental affect), “Climate change threatens our quality of life here in Germany as well.” (environmental affect), “Our way of life makes us responsible for many environmental problems in other countries as well (e.g. through the exploitation of raw materials or waste export).” (environmental cognition), Or “each and every individual bears responsibility for leaving a livable environment for future generations.” (environmental cognition).

#### 3.3. Seminar and student-specific statements

The lecturer offered various online workshops on the topic of climate change during the same period as the seminar. None of the students in either group had attended one of the workshops prior to the pretest. An insignificant number of the participants of the CG (1 of 21, p=0.306) stated in the posttest that they had attended this non-university offer. In the IG, the same question shows a significant increase with p<0.05 (6 of 21 participants in the posttest attended one of the workshops).

The other seminar- and student-specific statements examined in both surveys show no significant changes between the pretest and the posttest in either group (see table 5 (Tab. 5)). With average values between 3.65 and 3.86, both groups describe themselves in both surveys as not being more environmentally conscious than average (“Relatives and friends would describe me as very environmentally conscious.”).

The posttest-only statements show that, compared to the CG, the IG participants rated their knowledge about plastic consumption in the laboratory (“I know how to reduce plastic consumption in my daily laboratory routine.” p≤0.001), plastic as a health issue (“I know why plastic is a problem for health and environment.” p≤0.001) and the effects of climate change on health (“I feel well informed about the negative health effects caused by climate change.” p<0.05) as significantly higher. In contrast, a statement geared toward the content discussed in the CG shows a significantly higher agreement in the CG compared to the IG (“I feel well informed on the topic of organ donation.” p<0.05) (see table 6 (Tab. 6)).

#### 3.4. Effects on environmental knowledge

The CG and the IG showed a significant increase in knowledge with regard to their respective topics covered. The CG participants were able to significantly increase (p≤0.001) their knowledge with regard to general medical topics from an average of 4.50 (pretest) to 7.10 (posttest) (out of 10 possible correct answers). There was no significant increase in their knowledge about environmental topics. It increased from 3.14 to 3.29 (p=0.869).

**Figure 2:**
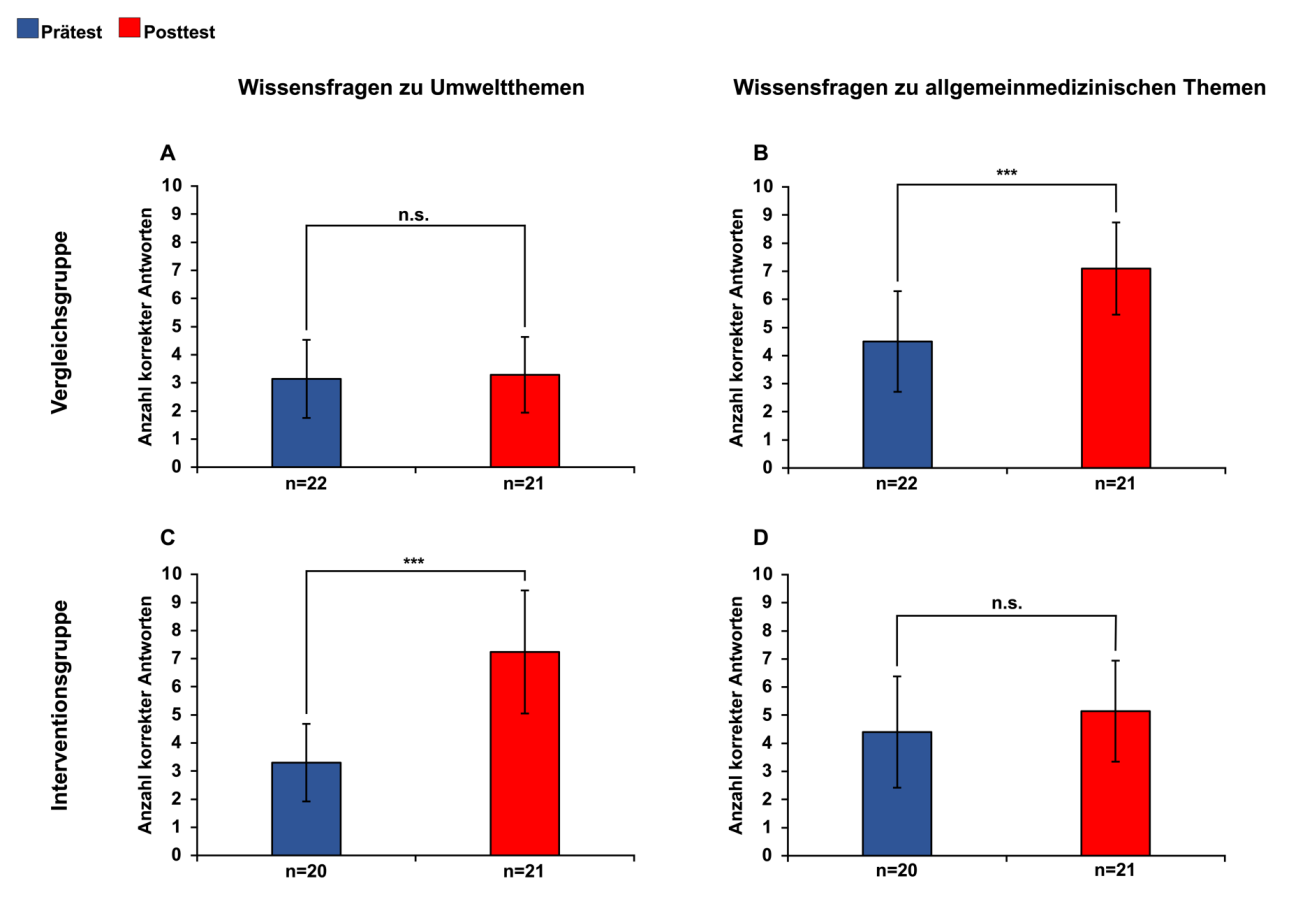
Knowledge question evaluation Maximum of 10 correct answers. p significance between pretest and posttest, n.s. not significant, ***p≤0.001

The IG participants achieved a significantly higher mean score for the environmental knowledge questions on the posttest (7.24) compared to the pretest (3.30; p≤0.001). There was no significant increase, 4.40 to 5.14 (p=0.234), with regard to the questions on general medical topics (see figure 2 (Fig. 2)).

## 4. Discussion

### 4.1. Environmental aspects relating to the future laboratory work of scientists in molecular medicine

Students of molecular medicine are preparing for careers in research-based medicine. As future medically oriented scientists with jobs in research institutions, universities and industry, laboratory work will be an important component of their work. Therefore, it is all the more important to create an awareness of the environmental impact of the health sector [5], [6] and research laboratories [7] from the start.

Accordingly, the presentation topics for the seminar were specifically tailored to the target group and their professional future, to ensure that the environmental communication that took place could have as lasting an effect as possible. By dealing with the medically relevant environmental topics, the students of the IG indicated on the posttest that, according to their own statement, they had significantly better knowledge about how to address these issues than the CG. This makes it easier for them to incorporate environmentally friendly working methods and also to pay attention to ecological effects in their professional lives.

#### 4.2. Increased interest in the climate and the environment due to the discussion of environmental issues

Despite the lack of significant effects in the categories of environmental affect, environmental cognition and environmental behavior, the intervention was able to increase the interest of some students in environmental issues. This is evidenced by the fact that several IG participants attended additional online workshops on environmental topics.

The fact that the knowledge of environmental topics was increased more significantly in the IG than the knowledge of general medical topics in the CG speaks for a fundamentally high interest of the students of molecular medicine in medically relevant environmental topics. The need for knowledge transfer in this area is evidenced by the significantly lower initial values compared to general medical topics. Despite extensive prior knowledge in general medicine across both groups, the IG showed greater increases in knowledge about environmental topics than the CG showed in general medical topics. In terms of factual knowledge of the topics covered in each group, the IG, with an average of 7.24 correct statements, even outperformed the CG with an average of 7.10 in the posttest. The increase in the environmental knowledge of the IG over the increase in the general medical knowledge of the CG illustrates the great interest in environmentally relevant content.

This observation is confirmed by a larger number of significant differences in seminar- and student-specific statements about the content offered to the IG compared to the content offered to the CG (see table 6 (Tab. 6)). This aligns with results that have shown that the communication of climate-related information at universities does not fail due to a lack of interest on the part of students, but rather due to a lack of expertise on the part of lecturers and a lack of support from the institutions [11]. For these reasons, environmental communication approaches should continue to be pursued and supported at the university level.

#### 4.3. Impact on the value-action gap reduction

The discrepancy between knowledge about the causes and consequences of climate change and any actual actions taken is a well-known problem (“value-action-gap” [14], [15], [16]). This phenomenon is evident on a global-political [12] as well as individual level [21]. The aim of this study was to determine whether this gap could be closed by hiding environmental and climate communication in an existing seminar.

On the one hand, the lower level of prior knowledge about environmental topics compared to general medical topics illustrates the need for knowledge transfer. The significantly increased factual knowledge about environmentally relevant content confirms the great interest in this area. On the other hand, no significant changes were observed in the environmental awareness subcategories. In particular, the changes to environment-related everyday behavior does not reflect the improved knowledge about environmental problems and the high approval rates for environmental affect and environmental cognition. Thus, at least in our seminar, the implicit teaching of medically relevant environmental topics in the hidden curriculum did not bridge this gap between knowledge and action.

In terms of climate psychology, the question arises as to why an awareness of climate change and its effects on society and health do not result in the corresponding environmentally conscious behavior. “Cognitive biases” are described as an explanatory approach [22]. In this context, information about climate change is filtered or not acknowledged in such a way that personal consequences or threats are ignored. In addition, the threat of climate change is not perceived as immediate or specific enough to make immediate action seem necessary.

Another approach is described by Bamberg and Möser, who show that environmentally conscious behavior correlates with self-interest, pro-social motives and moral norms [23]. In this context, awareness of a problem is to be regarded as a precondition. Even if knowledge of the negative consequences of global warming is present, as in the case of the students interviewed in the present study, other factors such as personal attitudes, norms and guilt are part of the decision-making process required to engage in sustainable behavior. In our approach to environmental communication, it was obviously not possible to address these aspects in such a way that they could have a sustainable influence on the students' environmental behavior.

Compared to study results from the German Federal Ministry for the Environment, Nature Conservation and Nuclear Safety (BMU) [17] and the Federal Environment Agency (UBA) [21], the results about the environmental awareness of the groups in the present study do not show any significant deviations. In the subcategories of environmental affect, environmental cognition and environmental behavior, the information provided by the students in the study is within the range of the representative information provided by the environmental awareness studies of 2018 and 2020. The high proportion of female participants, who tend to show more environmentally conscious attitudes [17], does not distort the picture of the results, as the gender distribution of the study participants also corresponds approximately to the distribution in the IG and CG. Due to the high response rate and the close correspondence between all seminar participants and the voluntary study participants in terms of socio-demographic characteristics, it can also be assumed that the group of molecular physicians studied is adequately represented.

Since the seminar was taught in an online format, the question arises as to how the results would have been, if the seminar had been taught in a traditional manner. Due to less direct contact and interaction between lecturers and students and the students among themselves, online teaching lacks an open exchange regardless of the curriculum [24], [25], [26]. There is strong evidence, however, that social interaction is closely associated with success in learning [27]. It is possible that deep-seated attitudes regarding the environment and climate could be influenced more sustainably in an in-person class.

#### 4.4. Hidden curriculum for teaching environmental topics

A hidden approach (hidden curriculum) was deliberately chosen in order to bring about environmental awareness changes by making a time- and resource-efficient adaptation of the existing seminar. The environmental topics were intentionally integrated into a course that teaches additive key competencies, since it was assumed that there was more free capacity here to address topics besides the actual curriculum content. The introduction of an additional learning objective or an additional learning event in the existing curriculum of the study program, which currently does not provide for a discussion of environmental and climate protection issues, was beyond our possibilities.

The results of this study suggest that an implicit engagement with environmental issues is not sufficient to lead to fundamental changes in the environmental awareness of students. Further research needs to show whether greater effects can be achieved with other teaching methods such as small group discussions or an active elaboration of the content. It becomes clear that “piggybacking” may be suitable as a starting point for the further development of broader curricula and interdisciplinary environmental education courses as well as for the transfer of knowledge about environmental content, but that it is insufficient as a sole initiative for increasing environmental awareness. More intensive teaching methods may be needed to counter the consequences of climate change with behavioral changes towards greater sustainability.

#### 4.5. Limitations of the study

In the present study, the small absolute number of participants in both groups represents a limitation with regard to the statistical power and significance of the data. In order to obtain a larger database, the survey could be repeated under identical conditions.

Only few statistically significant changes could be demonstrated in the study. One explanation besides the low intervention dose may be that the questions were not sufficiently adapted to the target group, so that minor changes in environmental awareness were overlooked. The timing of the posttest immediately following the final presentation may also influence the overall picture. As expected, factual knowledge is at its peak in the post-intervention period, whereas possible influences on environmental attitudes or actions may only become apparent later in the process. Altered results in terms of a loss of knowledge, increase in environmental awareness and changes in behavior cannot be ruled out in a time lag to an active engagement with the topic.

In connection with the high agreement values for the environmental awareness subcategories, a self-reporting bias must be assumed in part, which is rooted in the methodology of data collection by means of questionnaires and self-assessments. In this regard, study participants tend to distort the self-reporting to within the meaning of social desirability [28]. Assuming that the lecturer advocates a higher level of environmental awareness and could gain access to the answers, the participants may describe themselves as more environmentally aware than their everyday behavior reflects.

Another consideration relates to the learning motivation of the IG. They showed on average lower agreement values compared to the CG in connection with several statements (see 2. in table 5 (Tab. 5) and 1. - 6. in table 6 (Tab. 6)) and thus less interest in the seminar, the learning content and in environmental protection in general. Even if the statements do not show any significant differences between both groups, this confirms the lecturer’s impression.

#### 4.6. Lessons learned

This study shows that information about environmental and climate change issues in a hidden university curriculum is not sufficient to noticeably influence deeper environmental attitudes and everyday student behavior. In order to sustainably change the environmental awareness and behavior of students, future seminars might try to focus on a discussion of the content, not simply the preparation and presentation of the slides and might allow students to design their presentations in an independent manner. The lack of environmental awareness effects may be attributed to the mere optimization of the presentations and their slides. In the seminar described, however, this was one of the original learning objectives, which should not be changed.

Furthermore, the formulation of an explicit learning objective for environmental topics should be considered in future courses so that the respective issues can be addressed in detail. In order to further strengthen environmental awareness and to bring about more profound changes in sustainable behavior, environmental and climate topics probably need to be integrated more extensively and more broadly into the curriculum. As Molthan-Hill et al. call for, newly conceptualized curricula, modules, and transdisciplinary events should be created at the university level for this purpose, regardless of the degree program [12].

## 5. Conclusions

Despite a low intervention dose in this study, the environmental knowledge of the students surveyed was significantly increased. Although measurable effects on the reduction of the gap between knowledge and action were lacking, the engagement with environmental topics provided food for thought about sustainable designs in everyday research. In addition, some students became interested enough to participate in further climate workshops.

The purpose of illustrating this environmental communication approach in a university context was to serve as a good practice example and to encourage other lecturers to implement similar concepts tailored to their teaching content. Environmental and climate topics can be integrated with comparatively simple means, especially in courses that are ostensibly intended to impart additive key competencies.

## Acknowledgements

We would like to thank all those who were involved in this study for their support in developing the questionnaire, in particular Dr. Achim Schneider, as well as the Dean of Studies Office for assigning the groups and the students for participating in the surveys.

We would also like to thank Ute von Wietersheim (MBA , VW Translation Services) for the English translation of the manuscript.

## Competing interests

The authors declare that they have no competing interests. 

## Supplementary Material

Questionnaire

## Figures and Tables

**Table 1:**
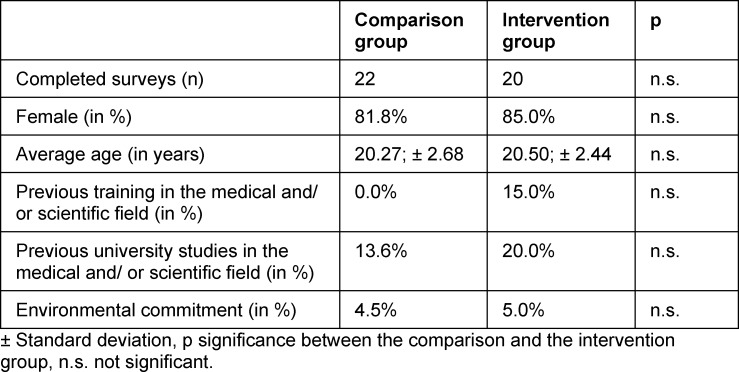
Sociodemographic data of the comparison and the intervention group from the pretest

**Table 2:**
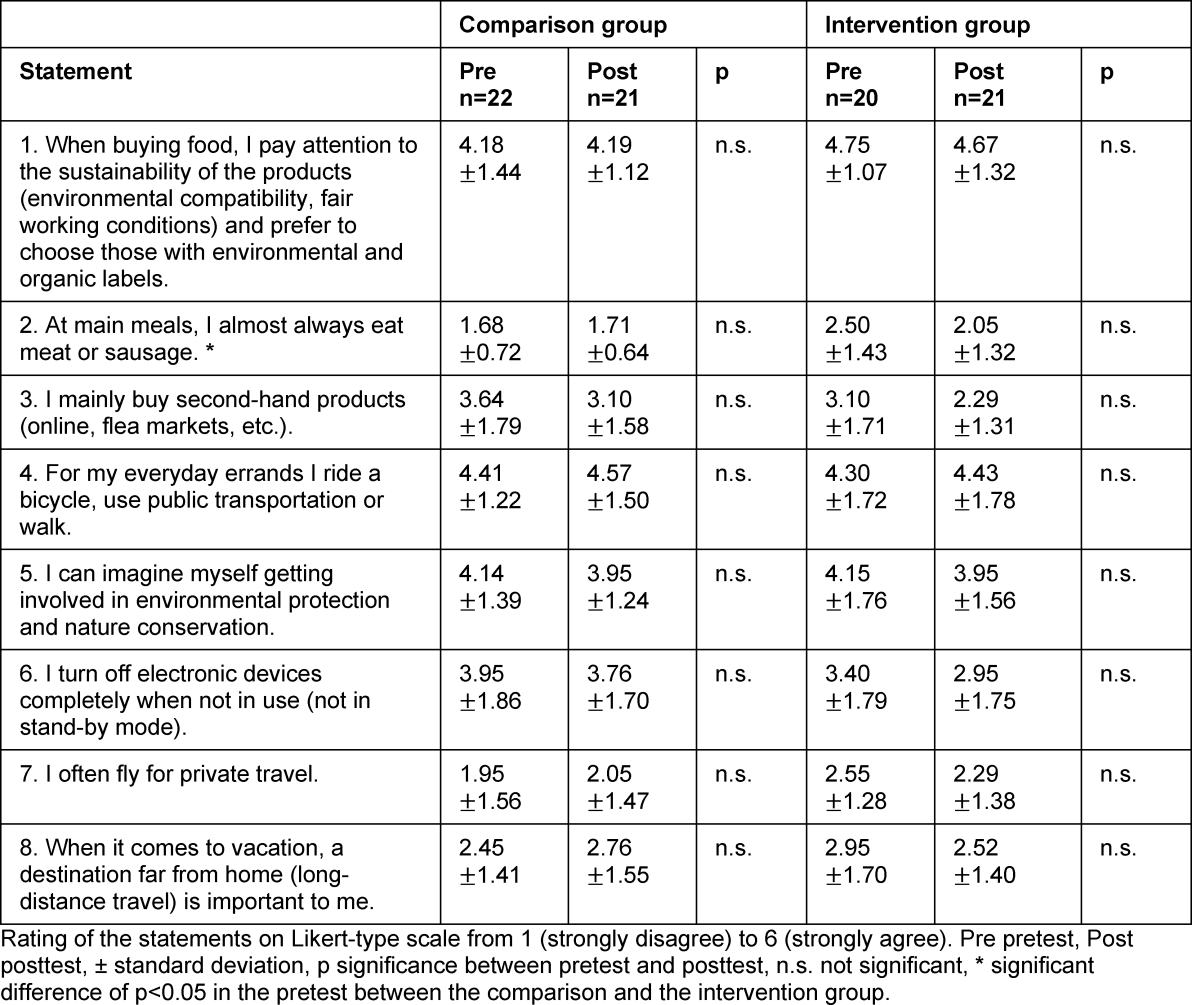
Evaluation of environmental behavior statements

**Table 3:**
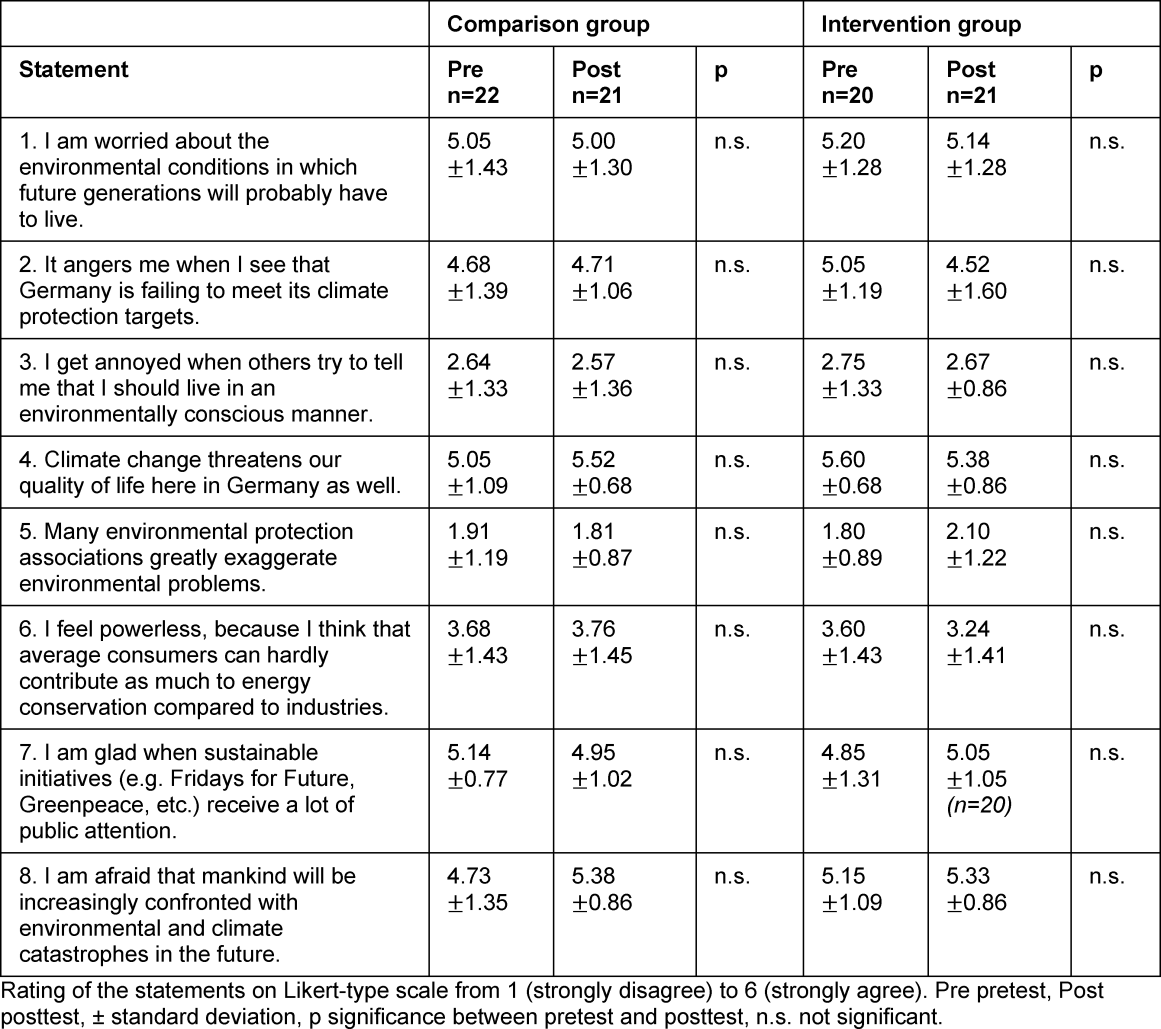
Evaluation of environmental affect statements

**Table 4:**
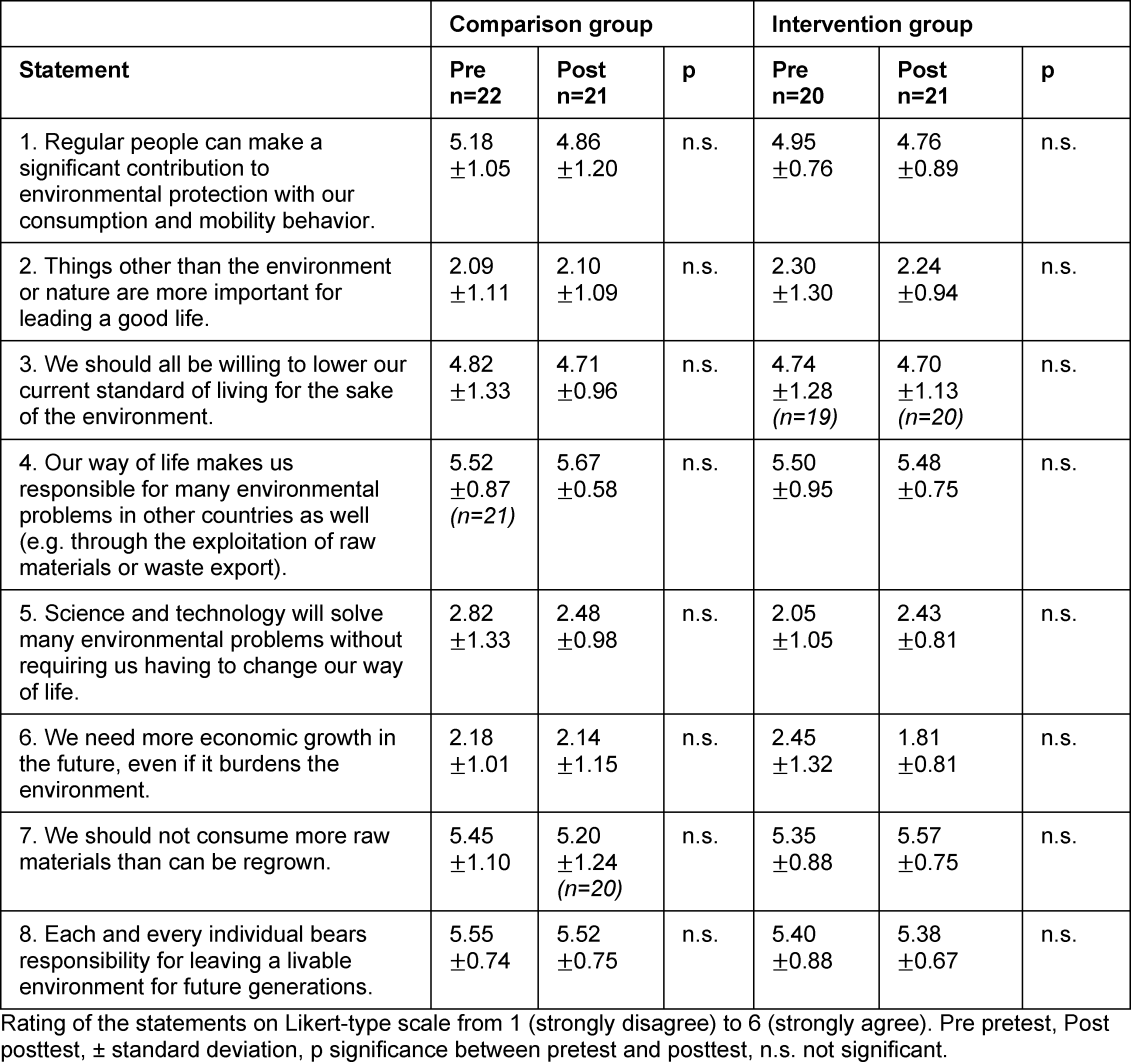
Evaluation of environmental cognition statements

**Table 5:**
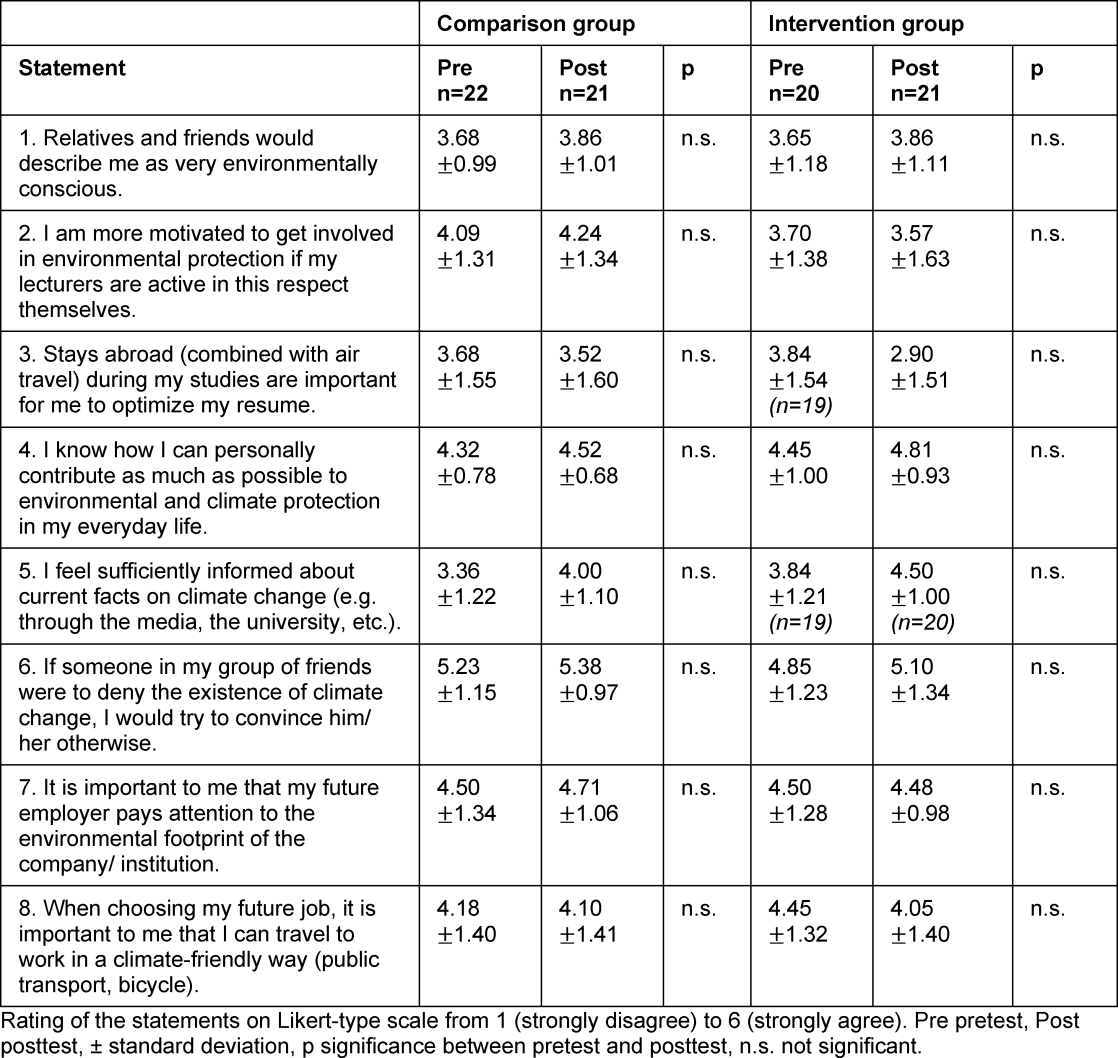
Evaluation of seminar- and student-specific questions in the pretest and the posttest

**Table 6:**
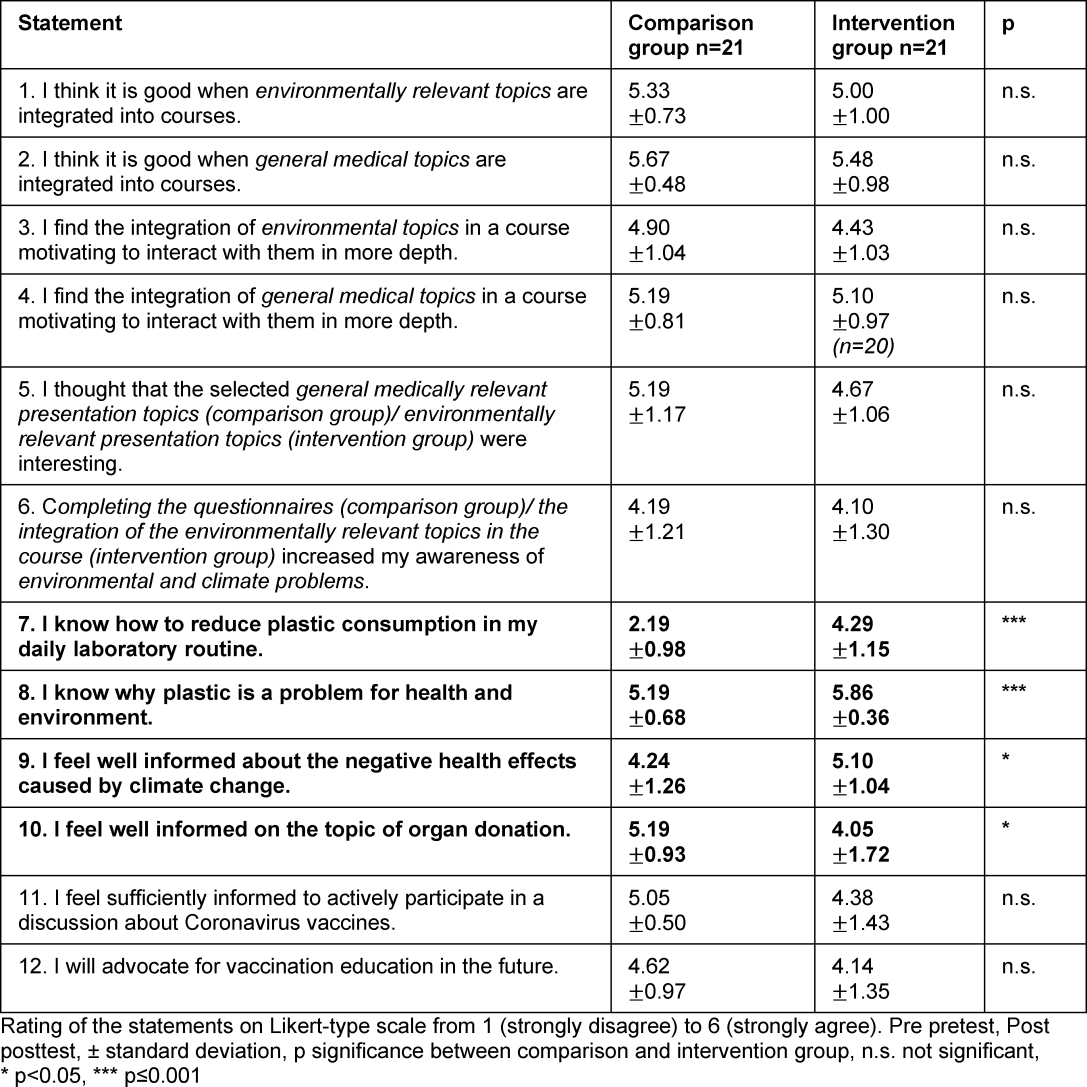
Evaluation of the seminar- and student-specific statements only on the posttest
