# Supplementary material for: A hidden curriculum for environmental topics in medical education: Impact on environmental knowledge and awareness of the students
Source: GMS J Med Educ. 2023 May 15;40(3):Doc27. doi: 10.3205/zma001609 (PMC10291344; doi:10.3205/zma001609)
Supplement: Questionnaire [file JME-40-27-s-001.pdf]

Attachment 1: Questionnaire

1 Sociodemographic data

Gender [1]
- female
- male
- other

Age (please click to select) [1]
- 17 – 45

I have already completed training in the medical and/ or scientific field (with/ without working in the profession and total duration > 1 year). [1]
- yes
- no

Prior to this study, I have attended other university studies in the medical and/ or scientific field (at least one year). [1]
- yes
- no

I am regularly committed (voluntarily) in the field of environmental issues (BUND, university group for sustainability of the University of Ulm, etc.). [1]
- yes
- no

2 Environmental affect

Please rate the following statements (agreement on Likert-type scale from 1-6 = strongly disagree - strongly agree)

1. I am worried about the environmental conditions in which future generations will probably have to live. [2]
2. It angers me when I see that Germany is failing to meet its climate protection targets. [2]
3. I get annoyed when others try to tell me that I should live in an environmentally conscious manner. [2]
4. Climate change threatens our quality of life here in Germany as well. [2]
5. Many environmental protection associations greatly exaggerate environmental problems. [2]
6. I feel powerless, because I think that average consumers can hardly contribute as much to energy conservation compared to industries. [1]
7. I am glad when sustainable initiatives (e.g. Fridays for Future, Greenpeace, etc.) receive a lot of public attention. [1]
8. I am afraid that mankind will be increasingly confronted with environmental and climate catastrophes in the future. [1]

3 Environmental cognition

Please rate the following statements (agreement on Likert-type scale from 1-6 = strongly disagree - strongly agree)

1. Regular people can make a significant contribution to environmental protection with our consumption and mobility behavior. [4]
2. Things other than the environment or nature are more important for leading a good life. [2]
3. We should all be willing to lower our current standard of living for the sake of the environment. [2]
4. Our way of life makes us responsible for many environmental problems in other countries as well (e.g. through the exploitation of raw materials or waste export). [3]
5. Science and technology will solve many environmental problems without requiring us having to change our way of life. [3]
6. We need more economic growth in the future, even if it burdens the environment. [2]
7. We should not consume more raw materials than can be regrown. [2]
8. Each and every individual bears responsibility for leaving a livable environment for future generations. [2]

4 Environmental behavior

Please rate the following statements (agreement on Likert-type scale from 1-6 = strongly disagree - strongly agree)

1. When buying food, I pay attention to the sustainability of the products (environmental compatibility, fair working conditions) and prefer to choose those with environmental and organic labels. [3]
2. At main meals, I almost always eat meat or sausage. [2]
3. I mainly buy second-hand products (online, flea markets, etc.). [1]
4. For my everyday errands I ride a bicycle, use public transportation or walk. [2]
5. I can imagine myself getting involved in environmental protection and nature conservation. [3]
6. I turn off electronic devices completely when not in use (not in stand-by mode). [4]
7. I often fly for private travel. [1]
8. When it comes to vacation, a destination far from home (long-distance travel) is important to me. [1]

5 Seminar- and student-specific questions

I have already participated in an event on "Climate, Environment, Plastics, etc." by Kühl/ Kühl. [1]

  yes
  no

Please rate the following statements (agreement on Likert-type scale from 1-6 = strongly disagree - strongly agree)

1. Relatives and friends would describe me as very environmentally conscious. [3]
2. I am more motivated to get involved in environmental protection if my lecturers are active in this respect themselves. [1]
3. Stays abroad (combined with air travel) during my studies are important for me to optimize my resume. [1]
4. I know how I can personally contribute as much as possible to environmental and climate protection in my everyday life. [1]

5. I feel sufficiently informed about current facts on climate change (e.g. through the media, the university, etc.). [1]
6. If someone in my group of friends were to deny the existence of climate change, I would try to convince him/ her otherwise. [1]
7. It is important to me that my future employer pays attention to the environmental footprint of the company/ institution. [1]
8. When choosing my future job, it is important to me that I can travel to work in a climate-friendly way (public transport, bicycle). [1]

6 Additional seminar- and student-specific questions only on the posttest (°variant for the comparison group, *variant for the intervention group).

Please rate the following statements (agreement on Likert-type scale from 1-6 = strongly disagree - strongly agree)
1. I think it is good when *environmentally relevant topics* are integrated into courses. [1]
2. I think it is good when *general medical topics* are integrated into courses. [1]
3. I find the integration of *environmental topics* in a course motivating to interact with them in more depth. [1]
4. I find the integration of *general medical topics* in a course motivating to interact with them in more depth. [1]
5. I thought that the selected *general medically relevant presentation topics*°/ *environmentally relevant presentation topics** were interesting. [1]
6. Completing the *questionnaires*°/ the *integration of the environmentally relevant topics in the course** increased my awareness of environmental and climate problems. [1]
7. I know how to reduce plastic consumption in my daily laboratory routine. [1]
8. I know why plastic is a problem for health and environment. [1]
9. I feel well informed about the negative health effects caused by climate change. [1]
10. I feel well informed on the topic of organ donation. [1]
11. I feel sufficiently informed to actively participate in a discussion about Coronavirus vaccines. [1]
12. I will advocate for vaccination education in the future. [1]

7 Environmental knowledge (multiple-choice format according to type A_{pos}, one correct answer out of five possible answers)

1. How many liters of water were used on average per day and bed in germany's hospitals in 2015? [1]
   a) 300-600
   b) 600-900
   c) > 1000
   d) 100-300
   e) < 100

2. For which component of the coronavirus does Biontech's mRNA vaccine encode? [1]
   a) Viral envelope
   b) Viral capsid
   c) Attenuated virus
   d) Entire viral genome
   e) Spike protein

3. Which of the sources indicated causes the most primary microplastics to enter the environment? [1]
   a) Waste and industrial companies
   b) Fiber wear from textiles
   c) Motor vehicle traffic
   d) Cosmetics and personal care products
   e) Plastic packaging

4. What are the current regulations for organ donation in Germany? [1]
   a) Dissent solution
   b) Extended consent solution
   c) There is no regulation on organ donation
   d) Decision solution
   e) Extended dissent solution
5. For which infectious disease is vaccination compulsory for children and staff in community and health care facilities throughout Germany? [1]
   a) Tetanus
   b) Hepatitis B
   c) Diphtheria
   d) Measles
   e) TBE

6. What percentage of emerging infectious diseases have an animal origin? [1]
   a) 20 %
   b) 5%
   c) 75 %
   d) 50 %
   e) 97 %

7. How many projects for the development of a vaccine against COVID-19 are there worldwide, according to the World Health Organization (WHO)? [1]
   a) 200
   b) ca. 75
   c) ca. 120
   d) ca. 40
   e) < 20

8. What was the estimated annual global plastic consumption in medical, biological and agricultural research laboratories in 2014? (in tons per year) [1]
   a) 5,5 M
   b) 12 M
   c) 3 M
   d) 0,2 M
   e) 20 M

9. What percentage of Germany's total CO2 emissions were caused by the healthcare sector in 2019? [1]
   a) <2%
   b) 9,2 %
   c) 5,2 %
   d) 18 %
   e) 12,7 %

Attachment 1 to Straßer P, Kühl M, Kühl SJ. *A hidden curriculum for environmental topics in medical education: Impact on environmental knowledge and awareness oft he students*. GMS J Med Educ. 2023;40(3):Doc27. DOI: 10.3205/zma001609

10. In which of the following products are most plasticizers used? [1]
    a) PET bottle
    b) Yogurt pot
    c) Sticking bricks
    d) PVC flooring
    e) T-Shirts

11. How much plastic waste was generated per person in germany in 2019? (private, commercial and industrial plastic waste combined) [1]
    a) 20 kg
    b) 76 kg
    c) 470 kg
    d) 205 kg
    e) 8 kg

12. How many of the 10 hottest summers since temperature records began (in 1881) have been recorded in the last 20 years? [1]
    a) 3
    b) 1
    c) 6
    d) 9
    e) 4

13. What is the share of patients who have already communicated with a doctor or therapist via video consultation (as of July 2020)? [1]
    a) 27 %
    b) 13 %
    c) 2 %
    d) 41 %
    e) 0,2 %

14. What amount of money did the German pharmaceutical industry spend on advertising in 2019? [1]
    a) 5 M €
    b) 7,8 B €
    c) 650 M €
    d) 1,5 B €
    e) 20 B €

15. Out of all the medical specialists, which ones use video consultations the most? [1]
    a) Surgical physicians
    b) Non-surgical physicians
    c) General practitioners
    d) Psychological-psychotherapeutic-psychiatric physicians
    e) Dentists

16. How many heart attack events could be prevented annually across Germany by curbing global warming from 3 degrees to 1.5 degrees? [1]
    a) 1000
    b) ca. 15000
    c) ca. 200
    d) 200000
    e) < 50

17. According to the German Foundation for Organ Transplantation, which organ was most common to be donated after death in 2018? [1]
    a) Lung
    b) Kidney
    c) Liver
    d) Pancreas
    e) Heart

18. How much did pharmaceutical companies spend on medical facilities and institutions in Germany in 2019 (e.g. for supporting events and donations)? [1]
    a) ca. 118 M €
    b) ca. 20 M €
    c) ca. 3 M €
    d) ca. 900 M €
    e) ca. 50 M €

19. How many EU countries (currently 27 member states) have mandatory vaccination for at least one disease? [1]
    a) 1
    b) 8
    c) 19
    d) 12
    e) 26

20. Which region of the world is most affected by declining populations of mammals, birds, amphibians, reptiles, and fish? [1]
    a) South and Central America
    b) Asia
    c) Europe
    d) North Amerika
    e) Africa

References

[1] Question designed by the authors.
[2] Rubik F, Müller R, Harnisch R, Holzhauer B, Schipperges M, Geiger S. Umweltbewusstsein in Deutschland 2018. Ergebnisse einer repräsentativen Bevölkerungsumfrage. Dessau-Roßlau (DEU): Umweltbundesamt, Bundesministerium für Umwelt, Naturschutz und Reaktorsicherheit; 2019.
[3] Scholl G, Gossen M, Holzhauer B, Schipperges M. Mit welchen Kenngrößen kann Umweltbewusstsein heute erfasst werden? Eine Machbarkeitsstudie. Dessau-Roßlau (DEU): Umweltbundesamt, Bundesministerium für Umwelt, Naturschutz und Reaktorsicherheit; 2016.
[4] Bundeszentrale für politische Bildung (Bpb). M 04.04 Musterfragebogen "Umweltbewusstsein und Klimaschutz in ...". Berlin: Bpb; 2007. Zugänglich unter/available from: https://www.bpb.de/lernen/grafstat/134897/m-03-04-musterfragebogen-umweltbewusstsein-und-klimaschutz-in-
